# Integrating GEDI and Landsat: Spaceborne Lidar and Four Decades of Optical Imagery for the Analysis of Forest Disturbances and Biomass Changes in Italy

**DOI:** 10.3390/s22052015

**Published:** 2022-03-04

**Authors:** Saverio Francini, Giovanni D’Amico, Elia Vangi, Costanza Borghi, Gherardo Chirici

**Affiliations:** 1 Department of Agriculture, Food, Environment and Forestry, University of Florence, 50145 Florence, Italy; saverio.francini@unifi.it (S.F.); elia.vangi@unifi.it (E.V.); costanza.borghi@unifi.it (C.B.); gherardo.chirici@unifi.it (G.C.); 2 Department of Bioscience and Territory, University of Molise, 86100 Campobasso, Italy

**Keywords:** GEDI, Landsat, lidar, regeneration, disturbance, harvest, biomass

## Abstract

Forests play a prominent role in the battle against climate change, as they absorb a relevant part of human carbon emissions. However, precisely because of climate change, forest disturbances are expected to increase and alter forests’ capacity to absorb carbon. In this context, forest monitoring using all available sources of information is crucial. We combined optical (Landsat) and photonic (GEDI) data to monitor four decades (1985–2019) of disturbances in Italian forests (11 Mha). Landsat data were confirmed as a relevant source of information for forest disturbance mapping, as forest harvestings in Tuscany were predicted with omission errors estimated between 29% (in 2012) and 65% (in 2001). GEDI was assessed using Airborne Laser Scanning (ALS) data available for about 6 Mha of Italian forests. A good correlation (r^2^ = 0.75) between Above Ground Biomass Density GEDI estimates (AGBD) and canopy height ALS estimates was reported. GEDI data provided complementary information to Landsat. The Landsat mission is capable of mapping disturbances, but not retrieving the three-dimensional structure of forests, while our results indicate that GEDI is capable of capturing forest biomass changes due to disturbances. GEDI acquires useful information not only for biomass trend quantification in disturbance regimes but also for forest disturbance discrimination and characterization, which is crucial to further understanding the effect of climate change on forest ecosystems.

## 1. Introduction

Forest ecosystems cover approximately one-third of Earth’s land surface [1]; they supply water, provide livelihoods, mitigate climate change, and are essential for sustainable food production [2,3]. Forests also represent a strong regulating element in global biogeochemical cycles and the climate system [4], sequestering up to 60% of the human-made carbon emissions in the atmosphere [5]. On the other hand, forests nowadays must also cope with the anthropogenic intensification of stressors that affect their condition, either directly through logging and clearing or indirectly through climate change, pollution, and invasive species [6]. Natural disturbances, such as fires, insect outbreaks, and windthrows are an integral part of ecosystem dynamics in forests around the globe. Usually, they occur as relatively rare events and form characteristic regimes of typical disturbance frequency, sizes, and severity over extended spatial and temporal scales [7]. However, forest disturbance regimes are increasing over time due to climate change. In particular, extreme drought events, wind storms, and forest fires represent the most relevant changes in Mediterranean forest ecosystems [8].

Remote sensing (RS) data have a long history in mapping forest disturbances. Optical sensors, in particular, have been widely used to automatically map forest disturbances across the globe at different spatial scales through several different approaches, including (1) bi-temporal analyses of image pairs [9,10,11], (2) time series segmentation [12,13], and (3) near-real-time monitoring of forest disturbances [14,15]. Results indicate that simple bi-temporal methods are not suitable for large-scale monitoring because they depend too much on data availability and imply calibrations that are difficult to generalize [4]. Similarly, time-series segmentation approaches—even if independent of data availability—require a set of parameters as input that should be calibrated explicitly for each ecosystem [16,17,18]. The accuracy of time-series segmentation methods also decreases for near-past detection applications [19]. Near real-time monitoring using optical remote sensing imagery instead is a relatively novel topic [2,20] and it is useful for quick assessment of relevant/extensive damages [21]. However, the achievable products often result in multiple inaccuracies, as the greater the promptness with which products are provided, the greater the expected number of errors in the maps [14]. To overcome these issues, an algorithm specifically conceived for Mediterranean ecosystems was recently developed (3I3D), which resulted particularly accurate in Tuscany [19], compared to more traditional approaches such as the Landrendr [12], the Global Forest Change map [22], and the Two Thresholds Method [21], which are three of the most sophisticated forest disturbance prediction algorithms. 3I3D was also recently used to map forest disturbance across Italy in 2018, and considerable accuracy was confirmed [20] as all the events in the reference dataset were detected - even with slightly smaller areas than the real ones.

While optical sensors are useful for mapping forest disturbances, they are less useful for predicting tree heights. Some studies have tried to predict the three-dimensional (3D) structure of forests from Landsat images [23] or integrated optical data with synthetic aperture radar (SAR) data (such as ALOS–PALSAR and Sentinel-1) [24]. Many studies have utilized modeling approaches to extend airborne laser scanning (ALS) data to communicate methods, outcomes, and accuracies and offer guidance on linking ALS metrics and ALS-derived forest attributes with broad-area predictors [25]. However, while the generation of spatially exhaustive lidar data would be useful for forest management purposes, lidar data predicted using satellite imagery are insufficiently accurate or reliable to drive decision-makers [26]. For these reasons, forests’ 3D structure is usually measured using ALS sensors [27,28,29], implying large survey and processing costs. As a result, updated information related to tree height is often not available wall-to-wall and over large areas but is constrained in both time and space. Consequently, estimates of the magnitude and distribution of aboveground carbon in Earth’s forests remain uncertain. On the other hand, knowledge of forest carbon content at a global scale is critical for forest management in support of climate mitigation actions. Consistent and large-scale monitoring of forest height is essential for estimating forest-related carbon emissions, analyzing forest degradation, and quantifying the effectiveness of forest restoration initiatives.

The Global Ecosystem Dynamics Investigation (GEDI) sensor offers unique opportunities to improve forest biomass mapping. It is the first space-based mission specifically conceived for retrieving the vertical structure of vegetation. GEDI was launched at the International Space Station in late 2018 and has been collecting unique data on vegetation structure since April 2019. GEDI products are provided at different preprocessing levels that allows the derivation of a variety of forest products, such as foliar canopy profiles, leaf area index (LAI), sub-canopy topography, and canopy height. GEDI promises to advance our understanding of forests. In one of the most groundbreaking works regarding the GEDI sensor, canopy height data were used together with Landsat imagery to create a 30 m spatial resolution global forest canopy height map for the year 2019 with RMSE ranging between 6.6 m—estimated using validation GEDI data—and 9 m—estimated using available lidar data [30]. Rishmawi et al. [31] integrated GEDI measurements with optical satellite observations, to produce maps at a 1 km resolution of US canopy cover (r^2^ = 0.79; RMSE = 0.09), plant area index (r^2^ = 0.76; RMSE = 0.41), foliage height diversity (r^2^ = 0.83; RMSE = 0.25), and tree height (r^2^ = 0.8; RMSE = 3.35 m). Leite et al. [32] presented a different usage of the GEDI data by predicting multi-layer fuel load in the Brazilian tropical savanna. On the other hand, most of the research focuses on simulated data despite the few studies we mentioned using GEDI data. For example, simulated data were used to compare biomass estimates from GEDI, NISAR, and ICESat-2 over California (Sonoma County), indicating greater precision in GEDI, with RMSEs of 57%, 75%, and 89%, respectively [33]. In the same study area, also using simulated data, Silva et al. [34] applied a multi-sensor (GEDI, ICESat-2, and NISAR) fusion approach to producing more accurate (RMSE of 52%) and spatially-complete aboveground biomass maps [33].

While the potential of the GEDI sensor still needs to be further explored, an additional GEDI product was recently released (Level 4) consisting of aboveground biomass density (AGBD) estimates for the period 18 April 2019 to 2 September 2020 [35,36]. To simplify the usage of GEDI AGBD data, an R package (GEDI4R) was recently developed [37] that provides functions for downloading, reading, clipping, exporting, and visualizing GEDI Level 4A data. Although GEDI Level 4 data is of great interest, to date, no studies exploiting these data have been developed. In particular, while one of the principal objectives for which the GEDI sensor was conceived is to “*provide answers to how deforestation has contributed to atmospheric CO_2_ concentrations*” (https://gedi.umd.edu/, accessed on 5 January 2022), currently, no study has analyzed the capability of GEDI to monitor biomass changes due to forest disturbances.

This study aims to assess GEDI AGBD data across Italy and its use to study the rate of biomass increase following forest disturbances, which is crucial considering that forest disturbances are expected to increase due to climate change [2,38]. First, we compared about four million AGBD data from GEDI pulses with ALS data available across Italy. Second, we mapped forest disturbances in Italy between 1985 and 2019 using Landsat imagery and 3I3D [20]. Subsequently, we analyzed about one million AGBD GEDI pulses for all years following predicted disturbances. A focus on coppice forest clearcuts was performed using a reference dataset of 10 Kha forest harvestings created in Tuscany between 1999 and 2016 [39].

## 2. Materials and Methods

### 2.1. Overview

This study focuses on Italian forests (Section 2.2) of which a large portion is covered by ALS data (Section 2.3). In Section 2.4, we introduce the Landsat data, the 3I3D algorithm, and how they were used to predict Italian forest disturbances for the period 1985–2019. A reference dataset of about 9500 ha of forest harvestings in Tuscany was also available (Section 2.4.1). Finally, in Section 2.5, we compared the biomass estimates provided by the GEDI sensors to ALS data (Section 2.5.1) and the years since the last predicted disturbance (Section 2.5.2).

### 2.2. Study Area

**Figure 1:**
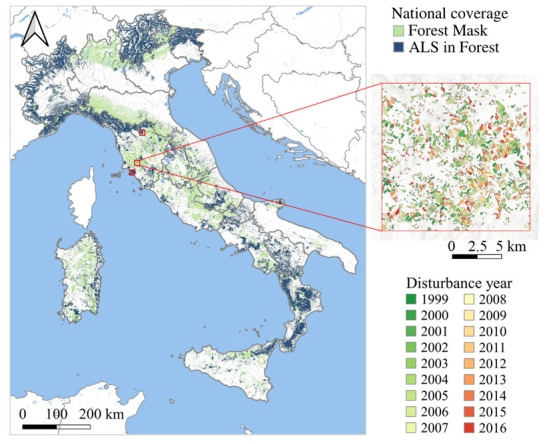
Study area, forest harvestings reference dataset (red boxes), and ALS data coverage.

The study was conducted in the approximately 11 million hectares of Italian forest land, as defined by the last national forest mask (Figure 1) [40,41]. A remarkable variability of vegetation characterizes the Italian peninsula due to a wide diversity of geographical and morphological conditions. Most forests are located upon hills and mountains [42], with broadleaf species covering about 68% of the total forest area. The most common tree species are, in order of frequency, Downy oak (*Quercus pubescens*), Pedunculated oak (*Q. robur*), Turkey oak (*Q. cerris*), Sessile oak (*Q. petraea*), and European beech (*Fagus sylvatica*), with each exceeding 1 million ha. The most common coniferous forests, especially in the Alps, are dominated by Norway spruce (*Picea abies*). Approximately 42% of Italy’s forests are managed as coppice forest [42], where clear-cut logging activities are conducted in rotations of 15 to 35 years on areas ranging from a few hundred m^2^ to 10–20 ha, but usually in the range of 1–5 ha [43,44].

### 2.3. ALS Data

ALS reference data were derived from available surveys collected in Italy from local, regional, and national authorities [40]. This national ALS dataset was constructed by harmonizing data from 29 flight campaigns carried out in the period 2004–2017, mainly between the years 2008 and 2011. ALS data was used to calculate a Canopy Height Model (CHM) that—to mimic the area of the Italian National Forest Inventory plots was aggregated at a resolution of 23 m. The national CHM covered 59% of Italian forests. Regionally (NUTS2), the percentage of forest area covered by ALS is not uniform. The CHM nearly or completely covered (>85%) the forest area of seven regions, i.e., Trentino-Alto Adige, Friuli Venezia Giulia, Basilicata, Aosta Valley, Piedmont, Calabria, and Liguria, while three regions had CHM coverage lower than 25% (Emilia-Romagna, Lombardy, and Lazio) [40].

### 2.4. Forest Disturbance Mapping

Data provided by the Landsat satellites constellations have long been used to conduct change detection analysis. The complete Landsat dataset (including Landsat 5, 7, and 8 missions) is available on Google Earth Engine (GEE), a cloud-based platform with high-performance or planetary-scale computing resources for processing vast geospatial datasets [45]. The long record of continuous measurement (since 1985), high spatial (30 m) and temporal (8–18 days) resolution, and near nadir observations [46,47,48] make Landsat imagery data one of the most valuable sources of information on forest disturbance detection [49,50,51,52,53]. On the other hand, cloud coverage [54] represents a limit to Landsat imagery implementation. In our study, we overcome this issue by applying the Best Available Pixel (BAP) composite procedure [55,56]. Pixel-based image compositing applies a set of user-defined rules to employ the extensive Landsat archive to generate cloud-free and radiometrically- and phenologically-consistent image composites that are spatially contiguous over large areas [57,58,59]. Specifically, the BAP allows one to select for each pixel location the “best” pixel among those available—thus from images that are not covered by clouds or shadows—based on a set of scores, among which are (i) the sensor score, (ii) the day of year score, (iii) the distance to cloud or cloud shadows score, and (iv) the opacity score. The BAP was recently implemented in GEE [60] with the full code openly available [61]. A detailed description of the application, guidance, and suggestions on BAP parameter setting is also provided on GitHub [62]. As a result of the BAP application, we obtained cloud-free composites for the whole of Italy for each year between 1985 and 2019.

Forest disturbance prediction was performed using the BAP composites and the GEE implementation [20] of the Three Indices Three Dimensions (3I3D) algorithm [19]. 3I3D is an unsupervised algorithm that predicts forest changes by analyzing the trends of three photosynthetic activity indices (3I) used as axes of three-dimensional space (3D) over three consecutive years. The photosynthetic activity indices are the Normalized Difference Moisture (NDMI) [63], the Normalized Burn Ratio (NBR) [64], and the Moisture Stress Index (MSI) [65]. For a detailed description of 3I3D, we refer to Francini et al. [19,20]. The complete 3I3D code is open access [66], for which further indications are provided on GitHub [67]. Thanks to the application of 3I3D, it was possible to obtain a set of 35 forest disturbance maps—one for each year between 1985 and 2019—with a spatial resolution of 30 m. 3I3D predict several different kinds of forest disturbance—including forest harvestings, forest fires, wind damages, insect attacks, drought, and frost damages—as long as they imply a decrease in the photosynthetic activity.

#### 2.4.1. Reference Data and Performance Assessment

A forest disturbance reference dataset [68] composed of approximately 9500 ha of forest clearcuts carried out between 1999 and 2016 was used to (i) study the rate of biomass increase following forest harvestings and (ii) estimate 3I3D performance in predicting forest harvestings. The reference dataset was obtained through photo interpretation within three study areas selected in Tuscany (Figure 1). The procedure proposed by Cohen et al. [69] was used. Each part of the dataset covered an area of 225 km^2^ [68]. Overall, the reference dataset consists of approximately 67 kha, mostly covered by forest. Inside the three areas, elevation ranges from 0 m above sea level (a.s.l.) to 1000 m a.s.l. Six out of the 14 European Forest Types are represented [70]. Within the regions composing the reference disturbance dataset, trees are cut with a rotation period of approximately 18–20 years. Since broadleaf forests are the most represented within the reference dataset, the coppice system is the dominant forest management scheme in these areas. Using this reference dataset, we evaluated the accuracy of maps by calculating (i) the number of true positives, corresponding to pixels correctly classified as disturbed; (ii) the number of true negatives, corresponding to pixels correctly classified as undisturbed forest; (iii) the number of false positives, corresponding to pixels incorrectly classified as disturbed; and (iv) the number of false negatives, corresponding to pixels incorrectly classified as undisturbed forest. Those parameters were then used to evaluate the maps’ omission and commission errors [71].

### 2.5. GEDI Level 4A Data

The GEDI sensor is installed on the International Space Station and is equipped with a geodetic-class laser altimeter/waveform lidar composed of three lasers that produce eight transects of structural information, providing 25-m resolution measurements of forest height in temperate and tropical forests (between 51.6° N and 51.6° S latitude). GEDI data are released with different products corresponding to different preprocessing levels [36]. In this study, we used the highest level of GEDI products, Level 4A, which predicts—in tons per hectare (t ha^−1^)—the aboveground biomass density (AGBD) for each sampled geolocated laser pulse [35]. AGBD predictions were derived from parametric models linking simulated relative height (RH) metrics from Level 2A to field plot estimates of AGBD. A calibration dataset of RH metrics and field estimates of AGBD were generated for multiple world regions and plant functional types (PFT) to train different models representing the combinations of regions and PFTs. With each footprint are associated a set of ancillary data such as uncertainty metrics, quality flags, and model inputs (land cover input data including PFTs and the world region identifiers). For details on the procedure used to generate Level 4A data, please refer to Duncanson et al. [36].

#### 2.5.1. Aboveground Biomass Density and Canopy Height Analysis

The correlation between AGBD and CHM data was assessed using all GEDI pulses acquired in Italy over areas where ALS data were available, resulting in 3,107,244 pulses. When the datasets are very large raw data, interpretation may be difficult, and data aggregation is needed [72,73]. For this reason, firstly, CHM and AGBD were ordered by CHM values and then aggregated by averaging into groups of size 25. Then, the coefficient of determination (r^2^) between aggregated AGBD and CHM was calculated [72,73]. The analysis was also repeated separately for each Italian region (NUTS2) to identify relevant differences in CHM-AGBD correlations among regions.

#### 2.5.2. Aboveground Biomass Density to Years since the Last Disturbance

To analyze the rate of biomass increase due to vegetation regrowth after forest disturbances, we compared AGBD estimations to Years since the Last Disturbance (YSLD), using (i) the 3I3D-predicted disturbances (Section 2.4) and (ii) the reference dataset of clearcuts performed in Tuscan coppice forests (Section 2.5). The expectation is that the greater the YSLD, the greater the AGBD. To perform this analysis, we selected all GEDI pulses acquired over predicted forest disturbances. Since GEDI data were available for years 2019 and 2020, and because forest disturbances were predicted for the period 1985–2019, possible YSLD values were from 0—a GEDI pulse acquired in 2019 over a forest disturbance predicted in 2019—to 35—a GEDI pulse acquired in 2020 over a forest disturbance predicted in 1985. To obtain results referring specifically to forest harvestings, we performed the same analysis using just the reference dataset (Section 2.4.1). As forest harvestings in the reference data occurred in 1999–2016, in this case, possible YSLD values were between 3 and 21.

## 3. Results

**Figure 2:**
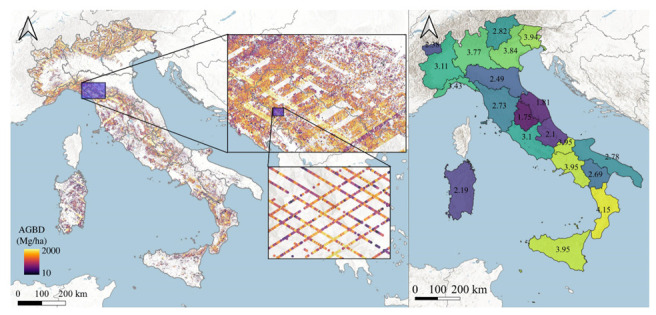
AGBD of GEDI pulses acquired over Italian forests. On the right is the percentage of GEDI coverage to the forest area by NUTS 2 unit.

A total of 6,381,454 GEDI lidar pulses were acquired over Italy between 2019 and 2020 (Figure 2). Among these, 5,167,420 pulses were in forests. The number of GEDI pulses per region ranged between 51,429 in Aosta Valley to 640,206 in Tuscany, while the number of pulses per km^2^ in the forested area ranged between 35.6 in Umbria to 84.4 in Calabria.

**Figure 3:**
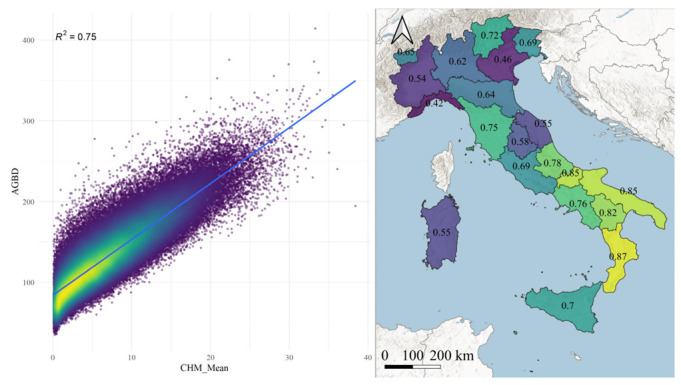
Left—scatter plot of aggregated AGBD and CHM with the blue line showing the linear correlation. Right—a map indicating the different correlations for each Italian region.

48% or 3,017,244 of the GEDI pulses acquired in Italian forests were over areas covered by ALS data. We found a consistent relationship between the AGBD and the CHM groups, with an r^2^ of 0.75, ranging between 0.87 in Calabria and 0.42 in Liguria (Figure 3).

**Figure 4:**
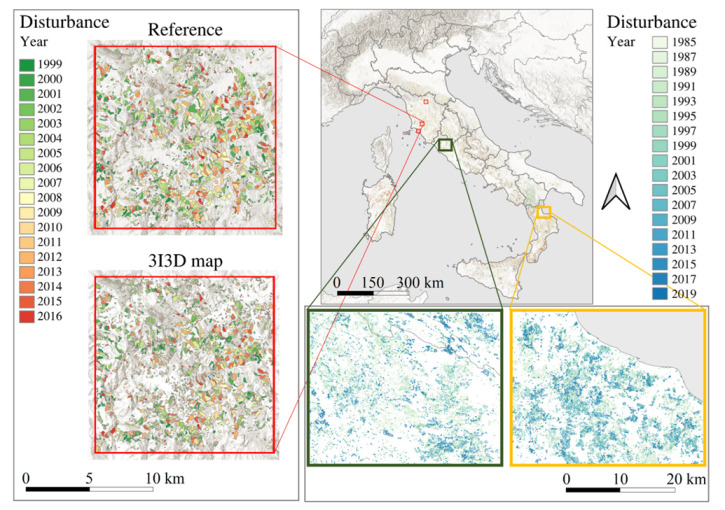
Comparison between predicted forest disturbance and clearcuts in one of three cells (in red) of the reference dataset (left). Predicted forest disturbance map (top-right) and two focuses (bottom-right).

**Figure 5:**
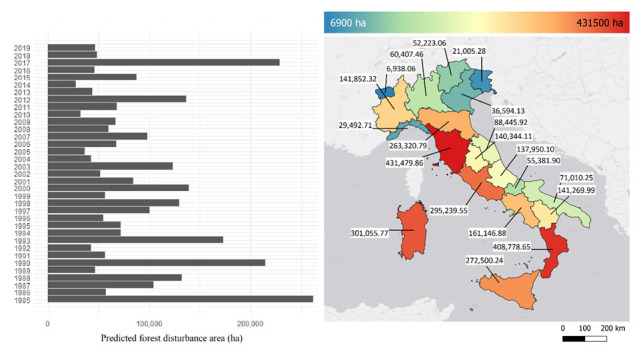
Per year area of forest disturbance predicted in Italy. On the right, the overall disturbance area is shown per region.

According to our automatic procedure (Section 2.4), 1,793,802 forest disturbances occurred in Italy between 1985 and 2019 (Figure 4). In terms of area, they covered approximately 3 million hectares, corresponding to 27% of the total Italian forest area. The average area of the predicted disturbance was 1.72 ha, while the median value was 0.83 ha. The years 1985, 2017, and 1990 were those with more forest disturbances, while the year 2014 was the less disturbed (Figure 5). At the NUTS 2 level, Tuscany was the most affected region, with 431,479.86 ha of forest disturbances during the study period. Overall, Tuscany, Calabria, Sardinia, Lazio, and Sicily host more than half (54%) of the total disturbed forest area. Using the forest harvestings reference dataset, estimates of omission errors ranged from 29% (in 2012) to 65% (in 2001), while commission errors were between 18% (2014) and 88% (2003).

**Figure 6:**
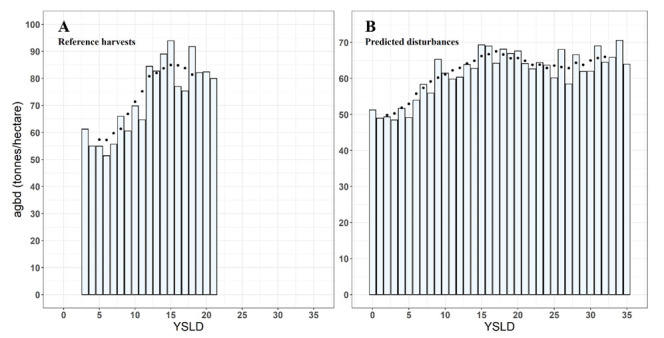
Median of AGBD values per YSLD in the reference dataset of forest harvestings (**A**) and all forest disturbances predicted across Italy (**B**). The black points show average values calculated using a moving window of 5 YSLD.

There were 1,080,483 GEDI pulses acquired over mapped forest disturbances. The number of pulses acquired yearly over forest disturbance ranged between 84,505 (for forest disturbances occurred in 2017) and 10,272 (for forest disturbances occurred in 2010). We found that the AGBD median values ranged between 48 t ha^−1^—on 3 YSLD—and 70 t ha^−1^—on 34 YSLD. Several oscillations in the AGBD values were recorded over time (Figure 6), but in general, AGBD in the reference forest harvestings (Figure 6A) increased more rapidly than in the predicted forest disturbance (Figure 6B), overtaking 80 t ha^−1^ in 11 YSLD. These results are congruent with the data provided by [74]. In both reference harvestings and forest disturbance, the AGBD does not increase after 15 YSLD. The average of the AGDB between 13 and 17 YSLD was 94 t ha^−1^ in harvested areas and 69 t ha^−1^ considering all disturbances.

**Figure 7:**
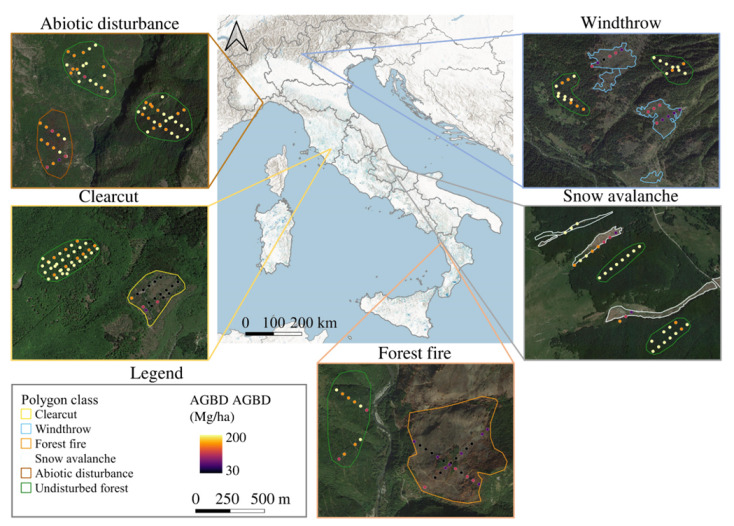
Comparison of AGBD between GEDI pulses acquired in 2019 over different kinds of forest disturbances that occurred in 2019.

Figure 7 shows AGBD values of GEDI footprints acquired over different kinds of forest disturbance and over undisturbed forest, suggesting relevant differences between undisturbed and disturbed forests, but also between the different kinds of disturbances.

## 4. Discussion

The three-dimensional structure of forests is an essential component for assessing changes in forest biomass due to human activities or natural disturbances. Structural data are also a key component in assessing forest habitat quality and biodiversity at local and regional scales [35], and assessing forests’ capacity to provide ecosystem services such as water regulation and soil protection [75].

Remote sensing holds a relevant part in forest monitoring, as disturbance maps can be predicted using automatic procedures and optical sensors images. Accordingly, in this contribution, we used Landsat BAP composites [55] and the 3I3D algorithm [19] to predict almost 2 million forest disturbances that occurred in Italy in the period 1985–2019. An in-depth performance evaluation of the 3I3D algorithm is presented in Francini et al. [19,20]. Commission errors reported in this paper are overestimated because 3I3D predicts several forest disturbance, while the reference dataset includes just forest harvestings. Similarly, as previously demonstrated [15,56], most omission errors coincided with forest disturbance correctly predicted, but with slightly smaller boundaries. To overcome this issue, a reference dataset of sample points may be preferred to polygons [20]. Either way, as expected, Landsat data provided crucial information on forest disturbances across the whole of Italy over a historical period of about four decades (Figure 4). The key years 1985 and 1990—characterized by very intensive frost events [76]—and 2017—characterized by several forest fires [77,78]—were correctly predicted as the years with the largest forest disturbance areas (Figure 5).

A well-known limitation of optical sensors is that they do not accurately capture biomass changes since they can measure spectral properties but not 3D forest structure. In this work, we demonstrated that the new GEDI mission and, most importantly, the very new GEDI AGBD product, provide global information that can consistently advance our understanding of forests and forest biomass changes. Accordingly, we found good correspondence (r^2^ = 0.75) between the groups of GEDI and ALS data in Italian forests. These results suggest that GEDI—even if not available wall-to-wall—provides data that may complement incomplete ALS datasets. GEDI data—with more than 6 million pulses acquired in 2019 and 2020—provided new information for 3% of Italian forests. In particular, the regions with the larger percentage of forests for which AGBD data were available were Calabria, Sicily, Campania, and Molise, with more than 4.0% of the forest area covered. Even for regions with the largest forest area, such as Tuscany and Sardinia, the percentage of forests with AGBD data were 2.7% and 2.2%, respectively. On the other hand, the regions with the smaller availability of ABGD data (<2.0%) were Umbria and Marche (Figure 2). Such data coverage is far from providing wall-to-wall information. However, combining GEDI pulses at different spatial scales—as is usually done with forest inventory data—may provide aggregated statistics of different forest variables, such as AGBD and forest height [79]. Future studies may further explore the usage of GEDI data in such directions since the percentage of forests covered by GEDI will still increase day by day, as long as GEDI continues to operate.

We showed that GEDI provides new data for quantifying biomass changes due to forest disturbances. Our results indicate that the AGBD product correctly captured the expected biomass increment due to post-disturbance forest recovery (Figure 6). Fluctuations of AGBD values over time were recorded as expected due to harvestings in different forest species or forests of different ages, and thus, with different biomass. Results also indicate the possible limitations of the GEDI sensor. In particular, in Figure 6A, two low AGBD values are visible corresponding with 16 and 17 YSLD, which coincides with a well-known drought event that occurred in 2003 in Italy and particularly affected coppice forests in Tuscany [80], to which the harvest dataset refers. The AGBD decrease captured by GEDI following 15 YSLD may be caused by a decrease in the vegetation photosynthetic activity and canopy condition related to the drought event, which may have resulted in a smaller amount of light reflected by the canopy resulting in the underestimation of the AGBD. This hypothesis is consistent with recent studies highlighting a major underestimation of dry forests’ carbon stocks through multi-sensor biomass mapping [81]. Considering all forest disturbances predicted across Italy, fluctuations are expected because 3I3D also predicts disturbances that do not imply biomass changes, the occurrence of which change significantly among different years (Figure 5, left panel). For these reasons, in this study - because of the lack of more detailed information on different forest disturbances - it was not possible to define a clear relationship between YSLD and AGBD. On the other hand, future studies combining GEDI and forest disturbance products may define a benchmark of biomass increase following different kinds of forest disturbance occurring in different forest types. Our results indicate that the GEDI sensor can provide data related to biomass changes due to forest disturbances, information that promises to advance our understanding of such events. For example, some of the disturbances we predicted using 3I3D (e.g., snow avalanches or abiotic disturbances) do not imply a significant loss of AGBD immediately after the disturbance, as is instead the case with logging and wind damage (Figure 7). For these reasons, GEDI may represent a game-changer not just for biomass monitoring, but also for forest disturbance characterization.

Our results also indicate that the maximum AGBD is reached at about 15 YSLD in harvests (Figure 6A) and other forest disturbances (Figure 6B). This peak appears earlier than expected. Such saturation may be related to the predominant forest coppice management. Coppice forests are characterized by a rapid biomass increase within the first 15 years after the disturbance [74]. Furthermore, according to forest regulation, standing trees that maintain forest cover are also released. This makes it difficult for the GEDI sensor to measure AGBD variations once the tree cover is re-established, even if it is fragmented [43,44].

Finally, an in-depth evaluation of AGBD to forest disturbance would require automatic forest disturbance characterization using remote sensing imagery [82]. On the other hand, it remains challenging to identify the disturbance type automatically [38], and—despite several attempts [2,56,59,83]—optical sensors do not allow good accuracies yet. This is especially true for Italian-managed forests, where natural disturbances are often superimposed by a management signal. An additional issue is that a precise, clear, and unique definition of forest disturbance is missing. For example, while FAO defines forest degradation as “the reduction of the capacity of a forest to provide goods and services’’, it is more than challenging to match this definition with forest disturbance products that can be derived with remotely-sensed data. On the other hand, such products are often the only ones available over large and remote regions of the globe. Furthermore, most ecologists tend to exclude natural disturbances from those which cause forest degradation [84], since natural disturbances can be considered part of forests’ life cycle. However, such alterations have the potential to profoundly impact the ability of forests to provide ecosystem services to society over the years. Similarly, climate-driven change in disturbance regimes could exceed the capacity of forests to regenerate. Even if forest ecosystems are well known for their high resilience capacity, this applies only to long-term changes in environmental conditions. Due to their long lifespan, trees are vulnerable to sudden changes because their ability to adapt rapidly is limited [8]. Therefore, changing natural disturbance regimes might have detrimental consequences for the functioning and services of Europe’s forests, and monitoring natural disturbances is thus of high importance.

## 5. Conclusions

Four main conclusions can be drawn as a result of the herein presented analysis.

First, optical sensors provide relevant information for wall-to-wall mapping of forest disturbance over large areas. Indeed, in this work, we used Landsat data and automatic procedures to monitor about 11 Mha of forests for the period 1985–2019, predicting almost two million disturbances.

Second, the GEDI sensor retrieves information on forest height and biomass—information not captured by optical sensors such as Landsat—and allows the identification of spatial and temporal trends regarding forest biomass changes that can be used to inform questions related to forest management and climate change. This study demonstrated that GEDI data—if properly aggregated—provide reliable information, as a good relationship (r^2^ = 0.75) with a large ALS dataset was identified.

Third, using the about two million forest disturbances we predicted, we demonstrated that the GEDI sensor provides useful information for monitoring biomass changes due to forest disturbance and, consequently, may assist in the future as a relevant data source for forest disturbance characterization.

Finally, while in this study it was not possible to use GEDI to statistically rigorously compare biomass change caused by different drivers of forest disturbance, future research should focus on this topic, which is presently needed. Indeed, forests represent a crucial component of the global terrestrial carbon cycle [85] and must take a part in the climate change fight. Due to climate change, forest disturbances are expected to increase and their capacity to absorb carbon may be altered. For these reasons, forest monitoring programs must consider a more in-depth characterization of forest disturbance in terms of biomass as well [86], which, as we showed in this paper, may require the integration of optical and space photonic sensors.

## Data Availability

For the data used please refer to: [66] for 3I3D dataset, [68] for reference dataset, [37] for GEDI data in Italy, [40] for Italian ALS data.
